# Supplementary material for: Using deep learning to quantify neuronal activation from single-cell and spatial transcriptomic data
Source: Nat Commun. 2024 Jan 26;15:779. doi: 10.1038/s41467-023-44503-5 (PMC10817898; doi:10.1038/s41467-023-44503-5)
Supplement: Supplementary file 1 — Supplementary Information [file 41467_2023_44503_MOESM1_ESM.pdf]

Supplementary Information for
Using Deep Learning to Quantify Neuronal Activation from Single-Cell and Spatial Transcriptomic Data

Ethan Bahl[1,2], Snehajyoti Chatterjee[3,4], Utsav Mukherjee[3,4,8], Muhammad Elsadany[1,2], Yann Vanrobaeys[2,3], Li-Chun Lin[3,4], Miriam McDonough[4,9], Jon Resch[4], K Peter Giese [5], Ted Abel[3,4], and Jacob J. Michaelson[1,3,6,7*]

[1]Department of Psychiatry, University of Iowa.
[2]Interdisciplinary Graduate Program in Genetics, University of Iowa.
[3]Iowa Neuroscience Institute, University of Iowa.
[4]Department of Neuroscience & Pharmacology, University of Iowa.
[5]Department of Basic and Clinical Neuroscience, King's College London, London, UK.
[6]Department of Biomedical Engineering, University of Iowa.
[7]Department of Communication Sciences & Disorders, University of Iowa.
[8]Interdisciplinary Graduate Program in Neuroscience, University of Iowa.
[9]Interdisciplinary Graduate Program in Molecular Medicine, University of Iowa.
* correspondence to jacob-michaelson@uiowa.edu

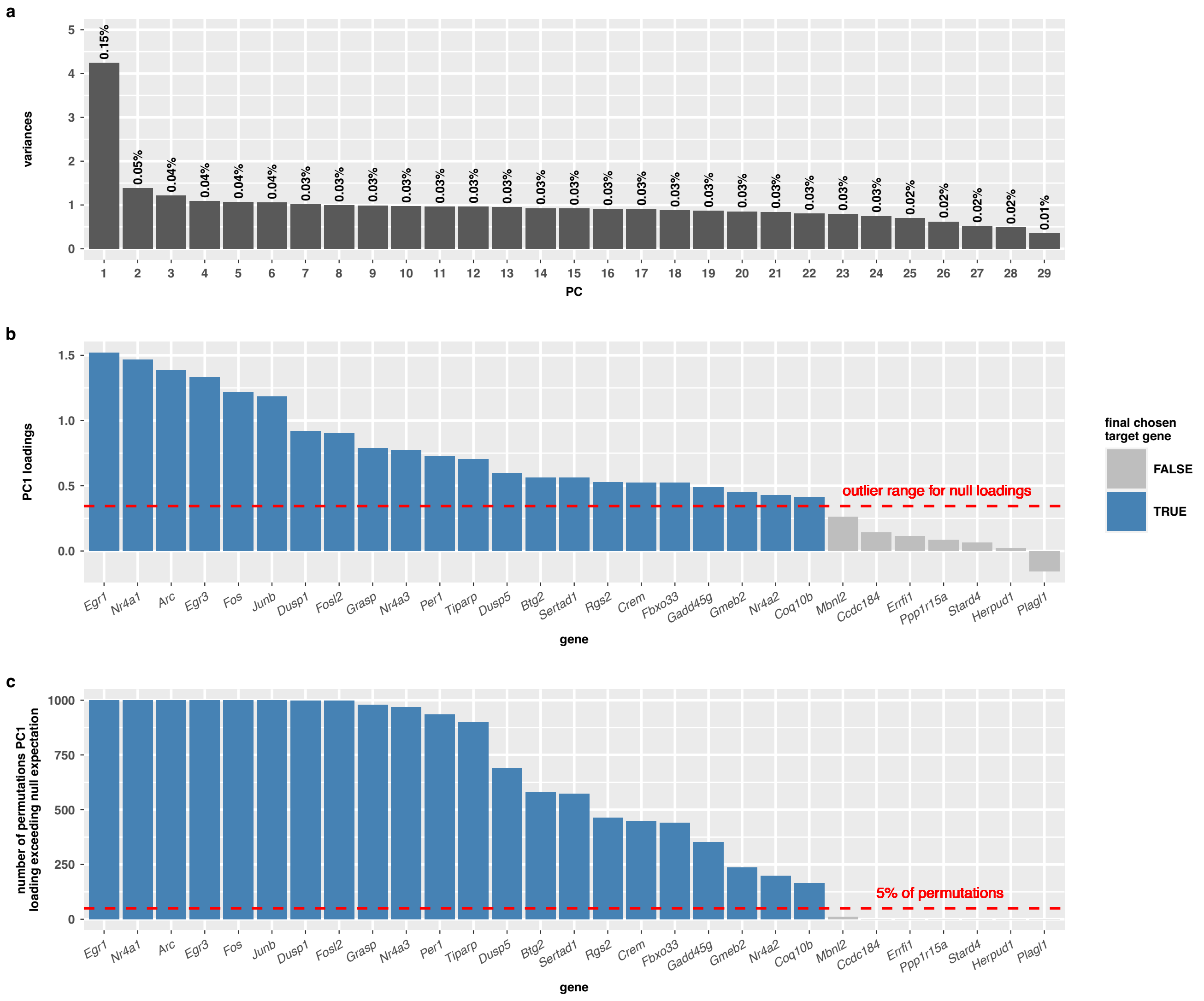

Figure S1 - Selection of target genes to be reconstructed by autoencoder. (**a**) A principle component analysis (PCA) of 29 candidate target genes reveals the dominance of the first component (PC1) over the other components, suggesting PC1 captures the majority of the underlying structure in the data. (**b**) Gene loadings onto PC1. The red line marks the outlier threshold for extreme loadings, as defined by a shuffled null PCA. (**c**) Number of bootstrap permutations where feature loadings of genes surpassed the outlier threshold in random subsets of samples. The red line indicates where loadings surpassed thresholds in 5% of permutations. Genes surpassing this threshold are colored blue, and these genes were selected as the final target genes used by the autoencoder. These findings show that the expression pattern of these genes is not overly complex and is suitable to be modeled as a single dimension. Source data are provided as a Source Data file.

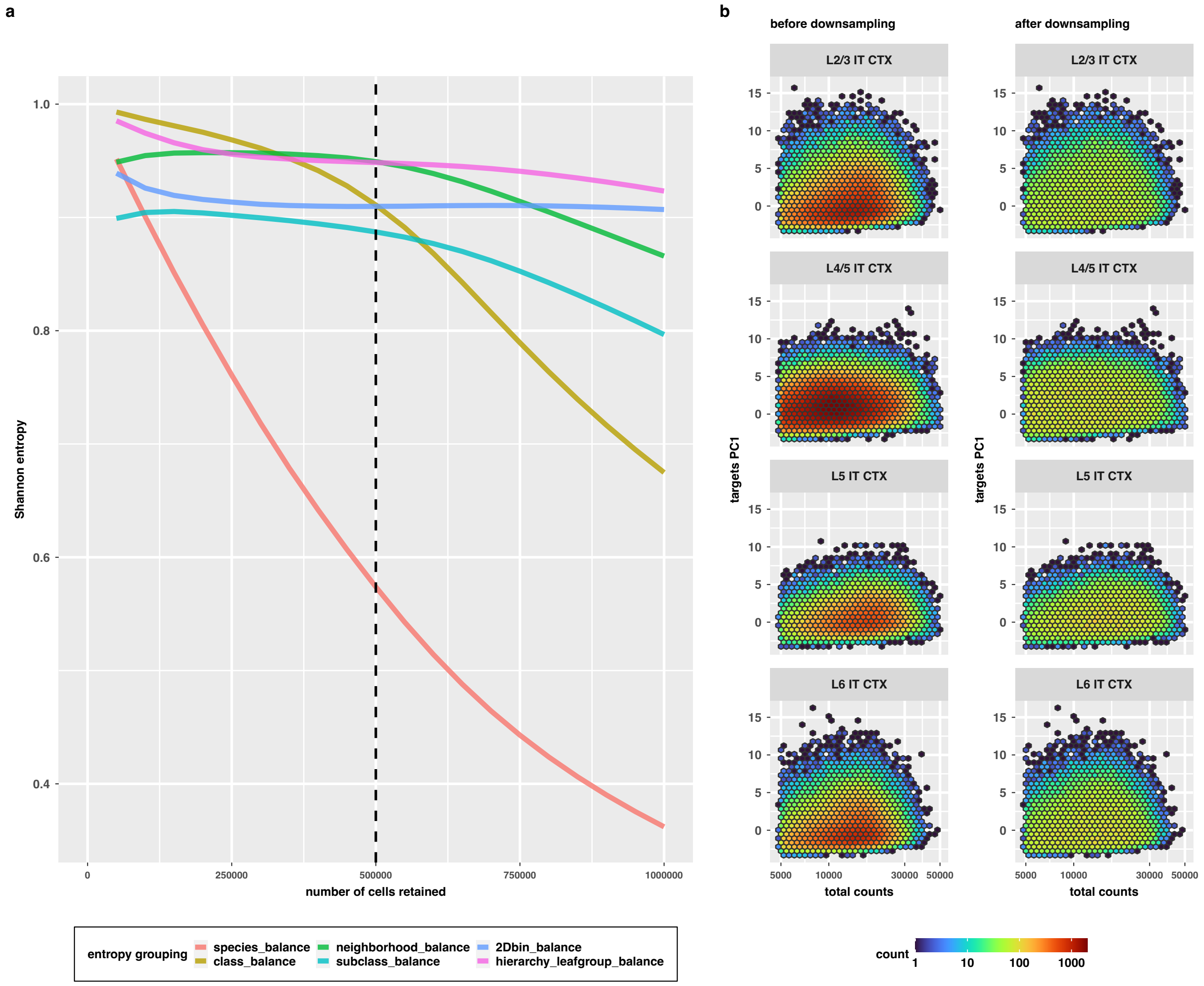

Figure S2 - Weighted downsampling improves balance and preserves heterogeneity. (**a**) Shannon entropy values (y-axis) measure balance of cell groupings across multiple downsampling thresholds (x-axis). Downsampling to 500,000 cells (dashed line) offers a balance of sample numbers and group balance, particularly for species (red), cell class (gold), and cell subclass (light blue). (**b**) Hierarchical downsampling visualized. Hexagon bin colors represents the number of cells in a given x,y coordinate range before downsampling (left) and after downsampling (right). The y-axis represents a cell's target gene PC1 score, and the x-axis shows total unique molecular identifier (UMI) counts per cell. Downsampling with hierarchical weighting preserves diversity in both biological and technical sources of variation. Source data are provided as a Source Data file.

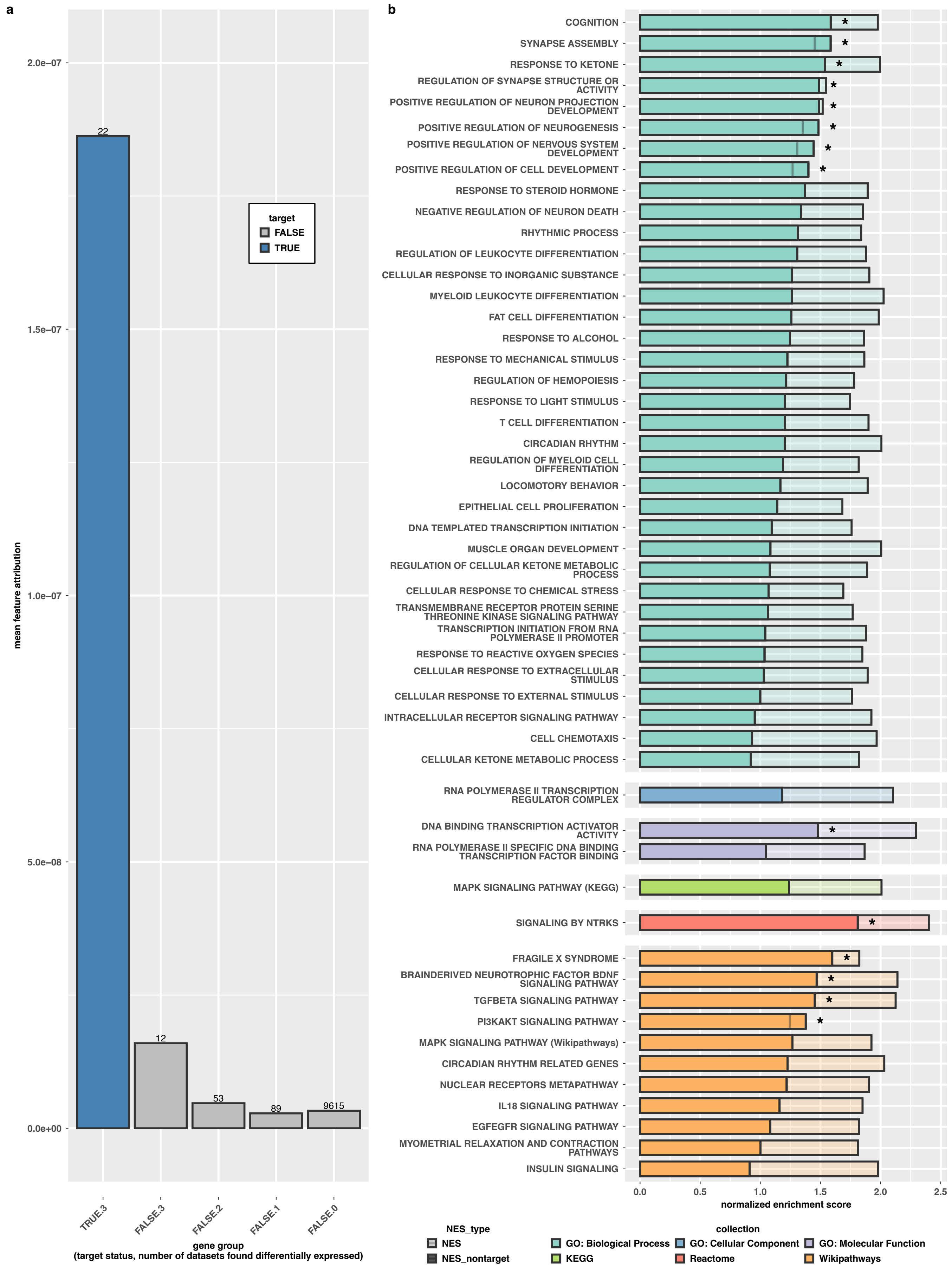

Figure S3 - Feature attribution of model predictions. (**a**) Mean feature attribution for target genes (blue) to non-target genes (gray), split by the number of stimulus-responsive differential expression lists they appeared in from the target selection analysis. The number of genes in each gene group are shown at the top of the bars. (**b**) Gene set enrichment analysis (GSEA) results for feature attributions. We examined positive enrichment scores (one-sided GSEA test implemented in the R package fgsea) for several gene sets using feature attributions of all input genes (light bar) or non-target genes (dark bar). Bars are colored by gene set collection. All gene sets displayed are significantly enriched after FDR-correction for multiple comparisons when considering attributions of all genes, and asterisks indicate the gene set remained significant without target genes included (see Supplemental Data 3). Source data are provided as a Source Data file.

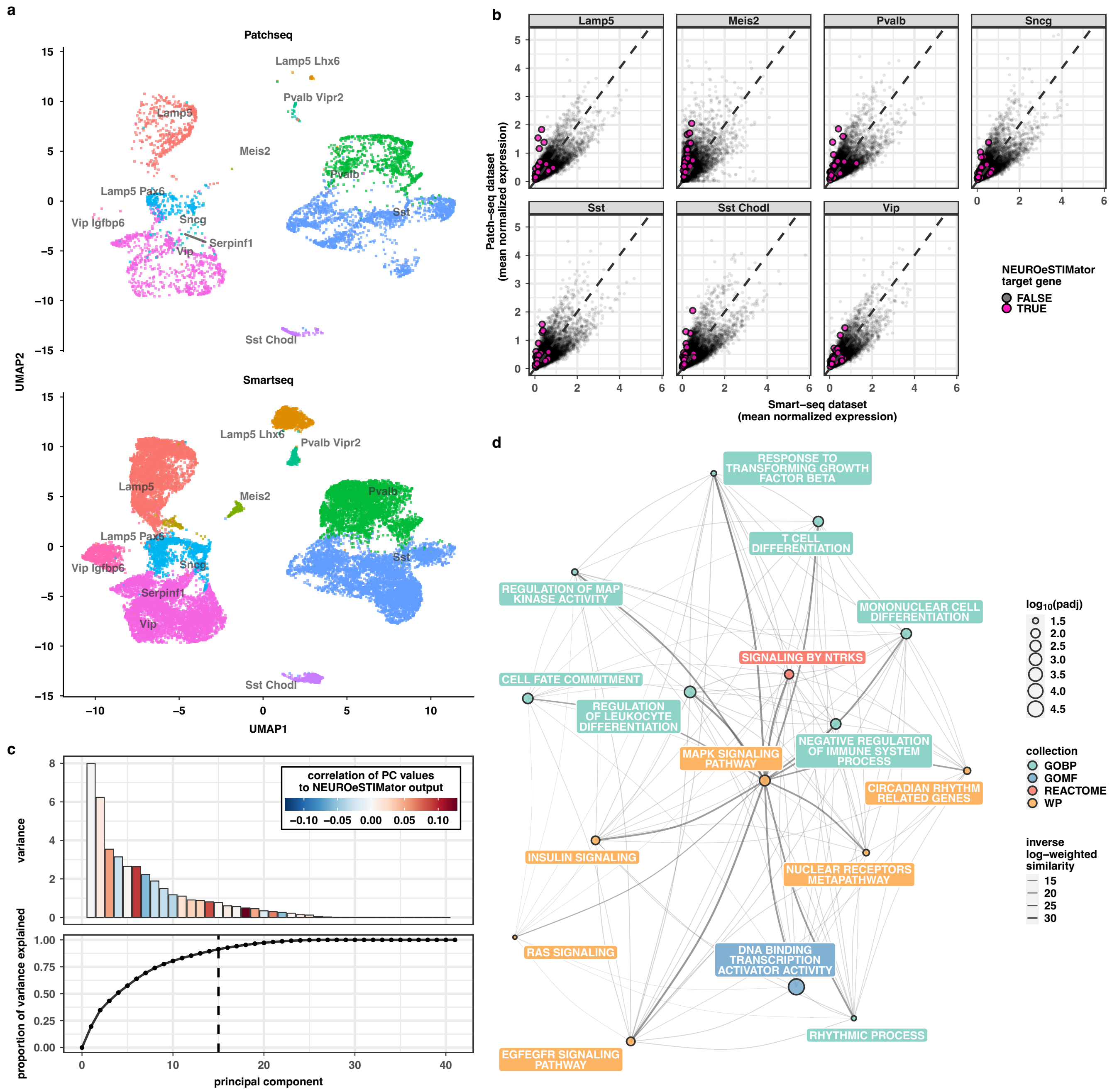

Figure S4 - Analysis of Patch-seq data with electrophysiological and transcriptional perspectives.
(**a**) Seurat integration of Patch-seq (top) and Smart-seq (bottom) datasets. Each dot represents a neuron and is colored by cell type. (**b**) Comparison of mean gene expression between Patch-seq and Smart-seq datasets. NEUROeSTIMator target genes are highlighted (pink) and the dashed line shows the y = x line. (**c**) Principal component analysis of electrophysiological features from the Patch-seq dataset. Principal component variances (top) are colored by the Pearson correlation between component values of cells and NEUROeSTIMator output. Positive correlations are colored red and negative are colored blue. Cumulative component contribution to total variance explained (bottom). The dashed line indicates where cumulative contribution surpasses 90% variance explained. (**d**) Network representation of a gene set enrichment analysis for gene loadings from canonical correlation analysis. Node size represents gene set significance (-$\log_{10}$ false-discovery adjusted p-values), node color indicates the source collection of gene sets, and edge width represents the inverse log-weighted similarity of gene sets. Abbreviations - GO: Biological Processes (GOBP), GO: Molecular Function (GOMF), Reactome, and Wikipathways (WP) (see Supplementary Data 4). Source data are provided as a Source Data file.

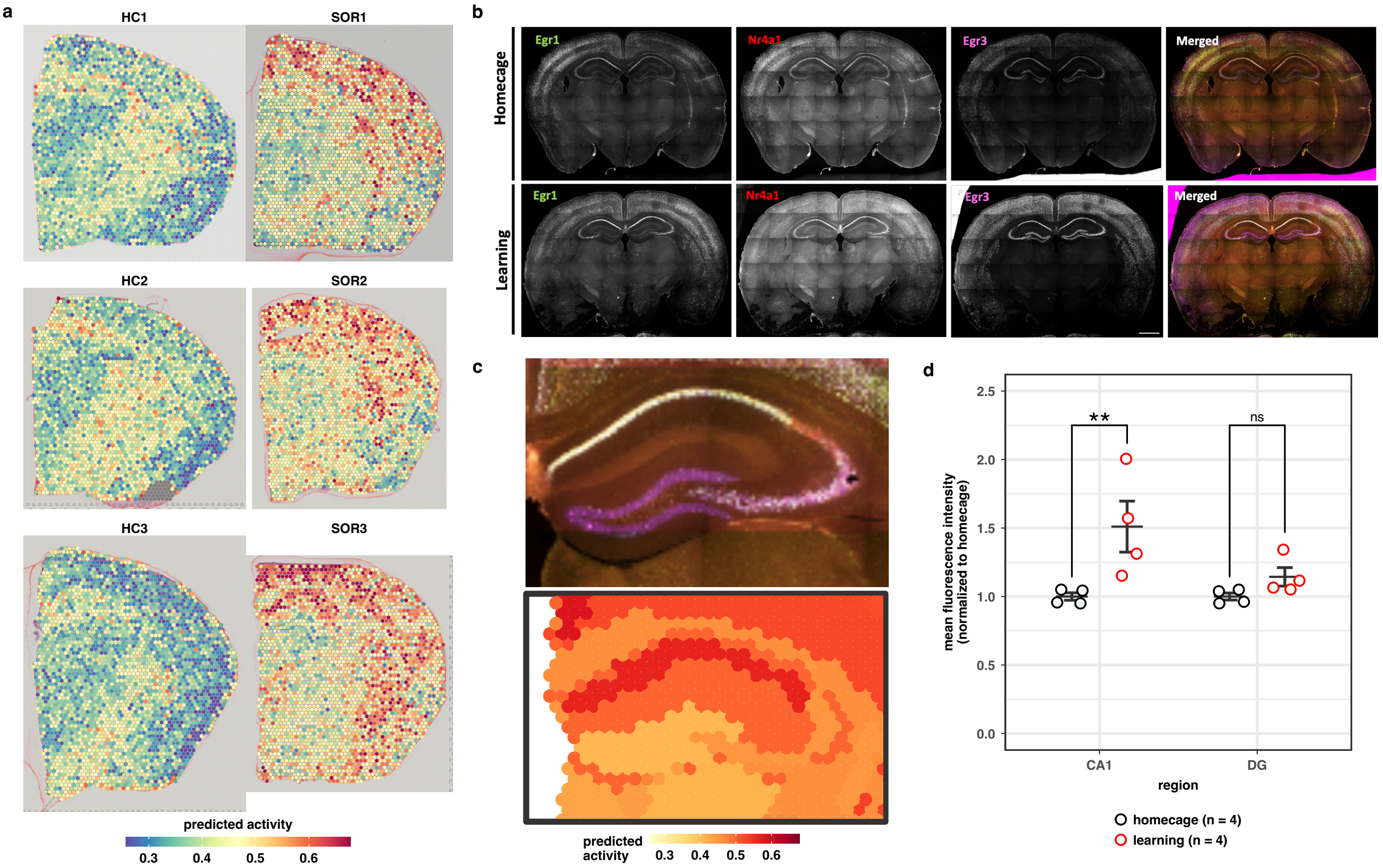

Figure S5 - Spatial transcriptomic techniques reveal region-specific activity in response to spatial learning.

(**a**) Predicted activity of individual Visium spots for brain sections from a homecage control group (left column, n = 3) and an SOR-trained group (right, n = 3). Red colors indicate higher activity and blue colors represent lower activity. (**b**) RNAscope validation of region-specific activation *Egr1*, *Nr4a1*, and *Egr3*. Scale bar represents 1000 um in length. Merged images (right column) shows overlap of probe fluorescence, with white colors representing regions of multi-probe overlap. (**c**) Image comparison of learning induced activity as measured by RNAscope (top) and Visium (bottom). (**d**) Dorsal hippocampus subregion-specific induction of transcriptional activity following SOR. Mean fluorescent intensity (MFI) of each RNAscope probe was averaged per mouse for both the CA1 (left) and dentate gyrus (DG, right) subregions of the hippocampus. Probe-averaged MFI was significantly increased in CA1, but not DG, after learning. Two-way ANOVA revealed a significant main effect of session (homecage vs learning): $F_{(1,6)} = 8.015$, $p = 0.0299$; Significant main effect of subregions (CA1 vs DG) $F_{(1,6)} = 6.636$, $p = 0.042$; Sidak's multiple comparisons tests: CA1 (homecage vs learning): $p = 0.0075$; DG (homecage vs learning): $p = 0.5553$. The experiment was performed twice in cohorts of two animals per group. A minimum of two replicate sections from each brain were quantified. Male mice, homecage (n = 4, black), learning (n = 4, red). Horizontal bars represent mean MFI, error bars represent ± SEM. *$p < 0.05$, **$p < 0.01$, ns: not significant. Source data are provided as a Source Data file.

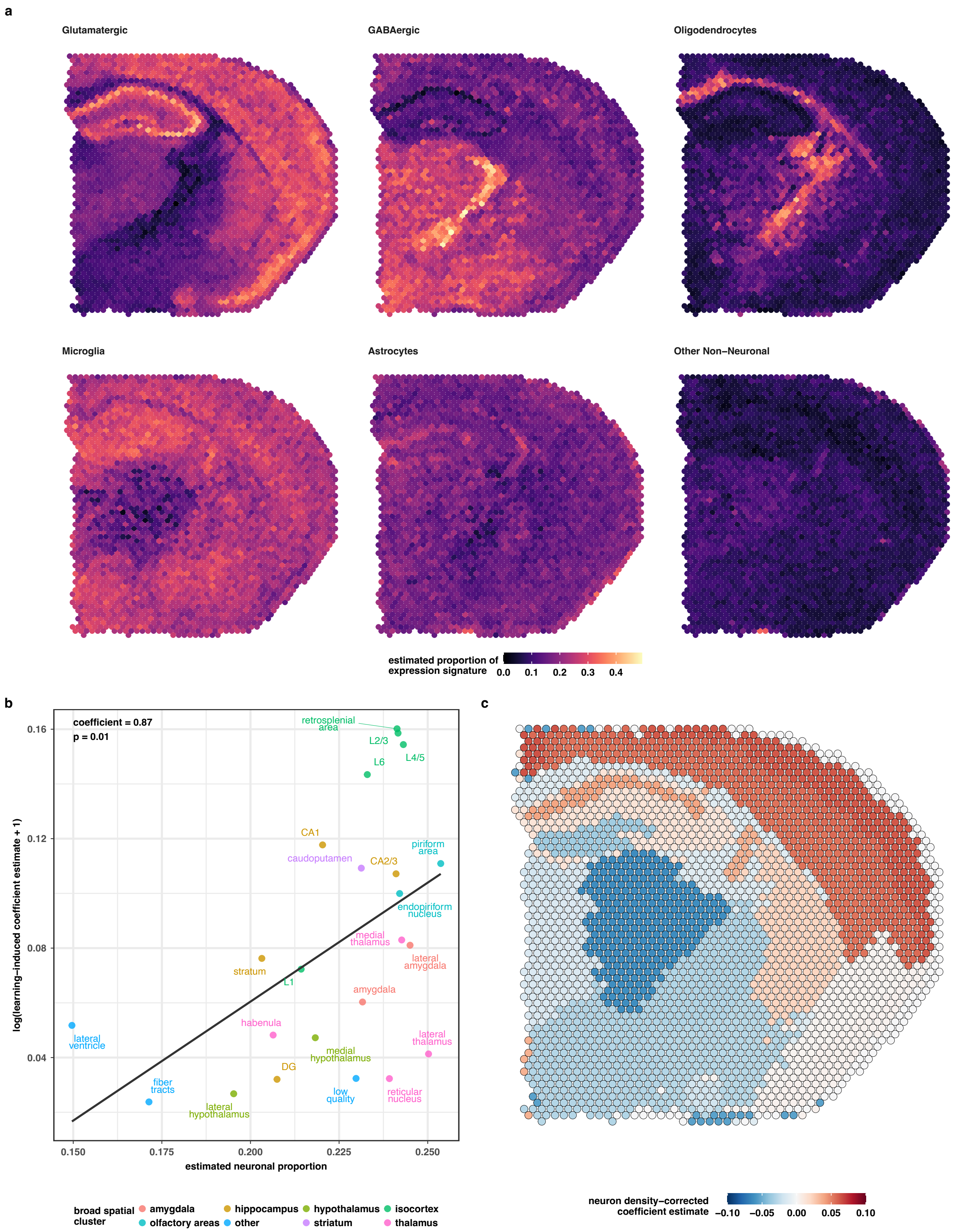

Figure S6 - Cell Type Deconvolution of Spatial Transcriptomic Data. (**a**) Results of cell type deconvolution in Visium samples, colored by estimated proportion of cell types. (**b**) Region-summarized relationship between estimated proportion of neuronal cell types and estimated effect of spatial learning (linear regression, coefficient = 0.87, $p = 0.01$, $n = 23$). Each point represents a brain region depicted in Figure 5a, and colors represent a broader spatial clustering. (**c**) Estimated effects of learning (see Figure 5c), adjusted for estimated proportion of neurons. Red indicates a coefficient greater than expected based on the trend observed in **b** (positive residual), while blue indicates a lower effect (negative residual) . Adjusted estimates are provided in Supplementary Table 7. Source data are provided as a Source Data file.

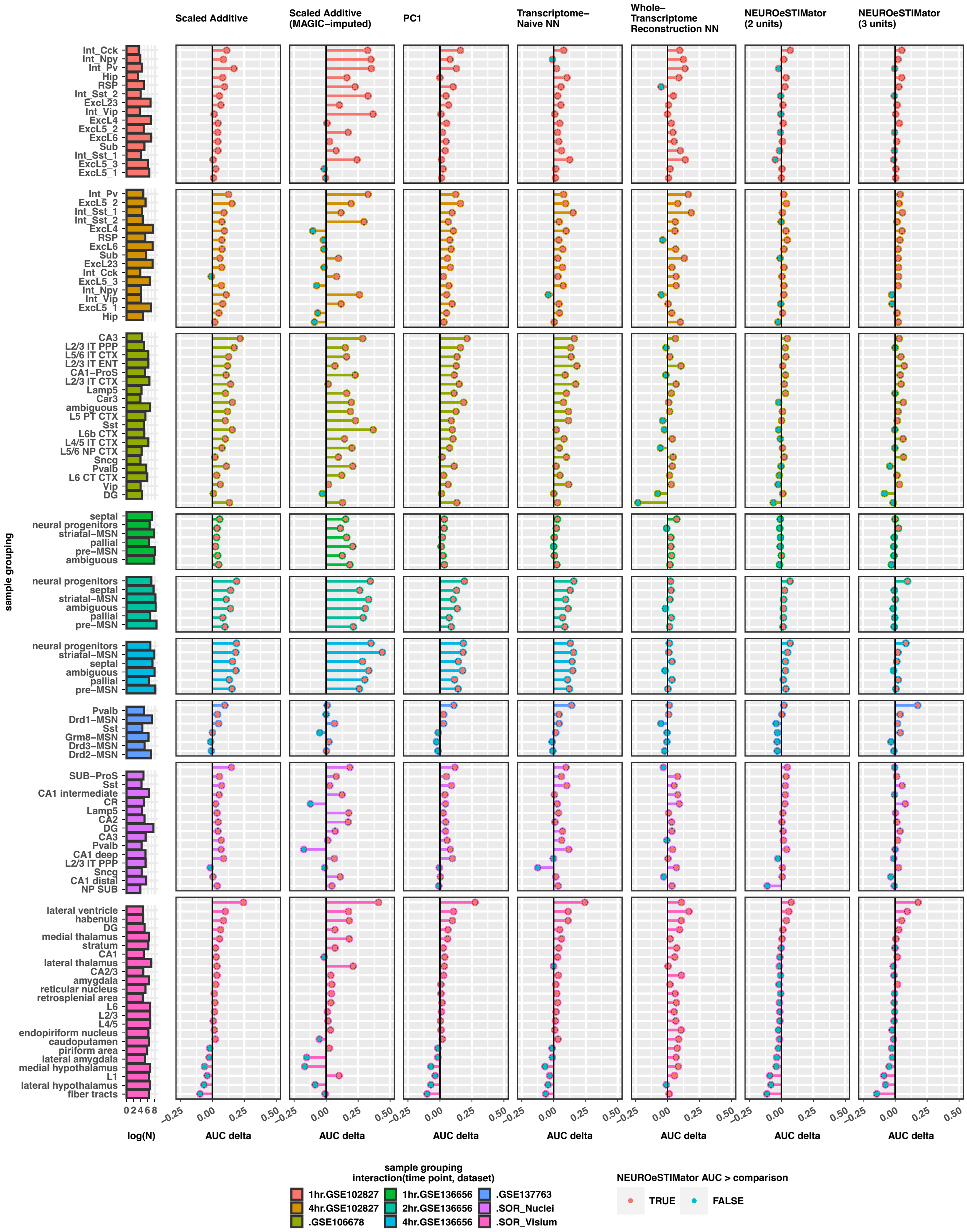

Figure S7 - Generalized Improvement in Classification Accuracy Over Competing Methods. Comparison of classification accuracy (AUC) of experimental stimulation groups for the NEUROeSTIMator model and seven competing methods (columns). AUC for separation of stimulated and unstimulated samples is calculated for NEUROeSTIMator and competing methods, and difference in AUC is shown on the x-axis. Positive x-axis values indicate NEUROeSTIMator classified the experimental groups with greater accuracy (points colored red) than the method being compared, and negative x-axis values represent reduced accuracy (points colored blue). Cells are grouped by dataset, cell type, and time point when applicable (rows), and these groupings are indicated by colors and row facets. Number of samples per comparison is shown on the left of the plot (log scale).
(See Supplementary Table 8).

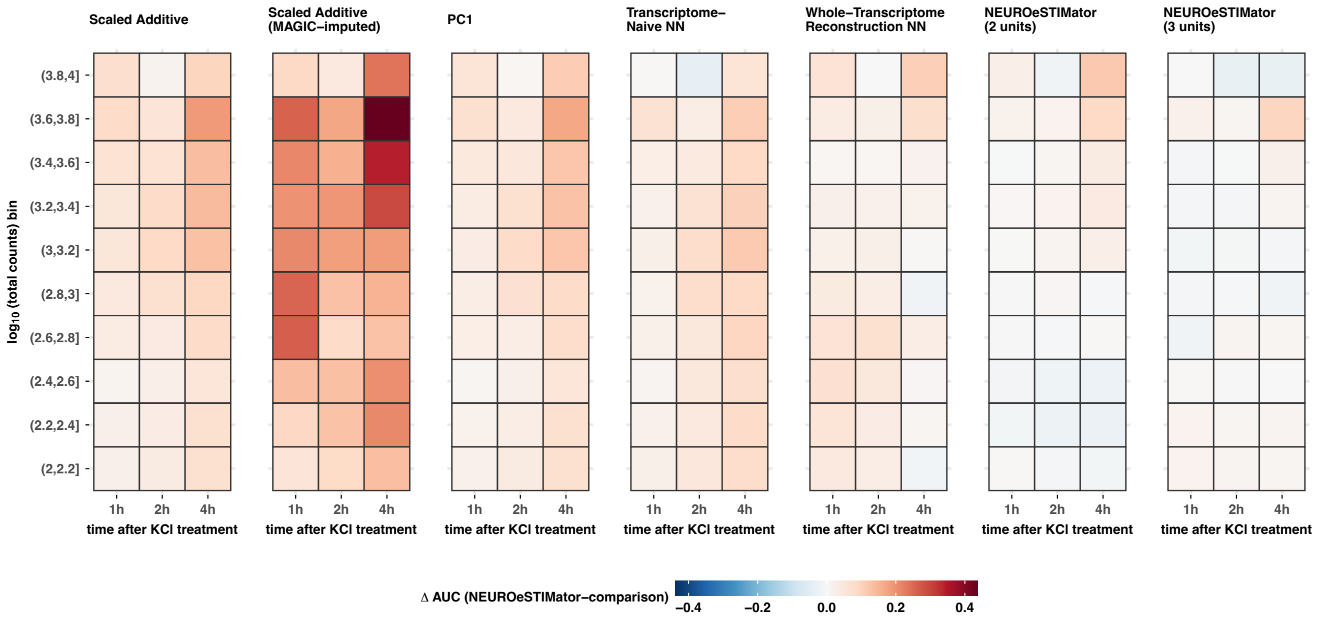

Figure S8 - Benchmarking performance across technical and biological variables. Comparing classification accuracy across technical and biological groups. Positive values (red) indicate NEUROeSTIMator classified the experimental groups with greater accuracy than the method being compared. Column titles indicate the method being compared, x-axis groups correspond to time passed since onset of stimulation in the KCl paradigm (GSE136656), and y-axis groups are binned by $\log_{10}$ sum of UMI counts. AUC values and grouping sample numbers are provided in source data. Source data are provided as a Source Data file.

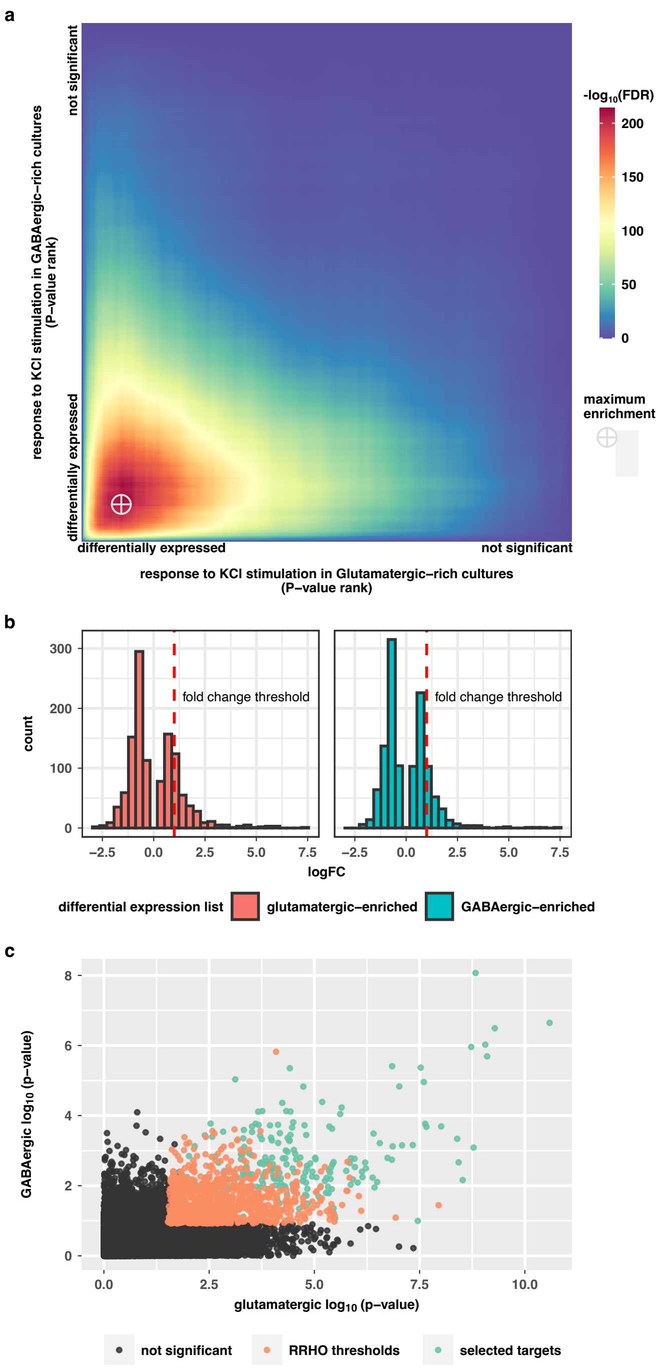

Figure S9 - Candidate Target Gene Selection. (**a**) Rank-rank hypergeometric overlap (RRHO) was used to identify p-value thresholds of maximum enrichment between differential expression lists for potassium chloride depolarization in glutamatergic-enriched and GABAergic-enriched neuron samples. The x-axis shows bins of 100 genes ranked by p-value from the glutamatergic samples, starting from most significant on the left. GABAergic results are shown on the y-axis with most significant genes starting from the bottom. Each cell of the heat map is colored by significance ($-\log_{10}$(FDR)) of the overlap between gene lists when considering the top genes given by the x-axis and y-axis values. Maximal overlap enrichment (designated by the crosshair symbol) was found at the intersection of the top 3,600 genes ranked by p-value for the glutamatergic results, and the top 3,300 for the GABAergic results (one-sided RRHO test, FDR = 3.05×10^{-215}), yielding a set of 1,184 overlapping genes. (**b**) A log fold change threshold of one was applied to both lists, further narrowing to a final set of 132 candidate targets from genes below RRHO p-value thresholds in both lists from this dataset. (**c**) Summary of the RRHO analysis showing significance of each gene (points) and the corresponding significance ($-\log_{10}$(p-values)) in the glutamatergic samples (x-axis) and GABAergic samples (y-axis). The 132 candidates selected are shown in green, while genes that passed RRHO p-value thresholds but not the log fold change thresholds are shown in orange.

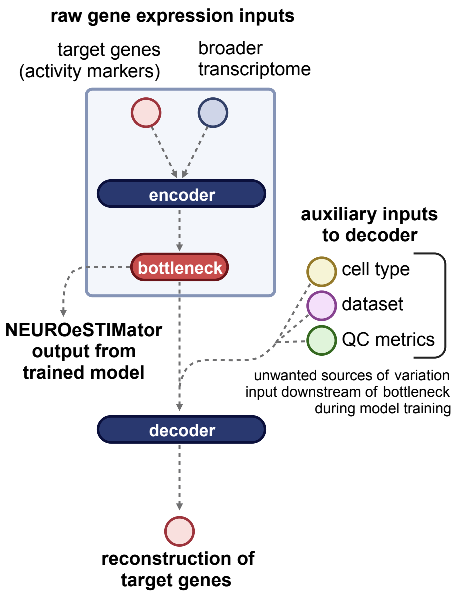

Figure S10 - Graphical representations of model architecture. NEUROeSTIMator was trained using an autoencoder that uses the whole transcriptome to reconstruct expression of neuronal activity marker genes. The transcriptome is condensed to a single-unit sigmoidal bottleneck layer before concatenation with auxiliary inputs of cell type, source dataset, and quality control (QC) metrics. The bottleneck output and the auxiliary information are used to decode the expression values of target genes. After training, NEUROeSTIMator consists of the encoder and bottleneck layer only, so auxiliary information is not needed for use. Fully detailed graphical representation of the model architecture is provided in source data. Source data are provided as a Source Data file.
