# Supplementary material for: Using deep learning to quantify neuronal activation from single-cell and spatial transcriptomic data
Source: Nat Commun. 2024 Jan 26;15:779. doi: 10.1038/s41467-023-44503-5 (PMC10817898; doi:10.1038/s41467-023-44503-5)
Supplement: Supplementary file 3 — Description of supplementary information [file 41467_2023_44503_MOESM3_ESM.docx]

**Description of Additional Supplementary Files**

File Name: Supplementary Data 1

Description: This table contains information about publicly available datasets used in this study. The table lists the dataset name, it’s use in this study, the species, the stimulation paradigm used, and the source tissue of the samples.

File Name: Supplementary Data 2

Description: This table contains gene-wise feature attributions for model predictions, as well as an indicator variable noting if the gene was a model target gene.

File Name: Supplementary Data 3

Description: This table contains data for the significant results from the gene set enrichment analysis (one-sided) performed on the feature attributions using the R package fgsea. There are columns containing the normalized enrichment score and false discovery rate (FDR)-adjusted p-values from the analysis applied to all genes (padj.all, NES.all), as well as the analysis applied only to non-target genes (padj.nontarget, NES.nontarget).

File Name: Supplementary Data 4

Description: This table contains data for the significant results from the gene set enrichment analysis (one-sided) performed on the gene loadings from the canonical correlation analysis of the Patch-seq data.

File Name: Supplementary Data 5

Description: This table contains results for the Kolmogorv-Smirnov (KS) tests (one-sided) shown in Figure 3a-c.

File Name: Supplementary Data 6

Description: This table contains results for the KS tests (one-sided) for the visual cortex neuron dataset in response to light exposure.

File Name: Supplementary Data 7

Description: This table contains results for the Visium dataset shown in Figure 5d and Supplementary Figure 6. For each anatomical clustering region, results of the linear regression are shown, in addition to cell type deconvolution estimates of cell types and the neuron density-corrected coefficient estimates.

File Name: Supplementary Data 8

Description: This table contains the performance benchmarking results shown in Supplementary Figure 7 where NEUROeSTIMator is compared to several other approaches to quantifying activity. Results are given as area under the curve (AUC) for separating stimulated samples from unstimulated controls, split by dataset and neuron type.
