# Supplementary material for: Using deep learning to quantify neuronal activation from single-cell and spatial transcriptomic data
Source: Nat Commun. 2024 Jan 26;15:779. doi: 10.1038/s41467-023-44503-5 (PMC10817898; doi:10.1038/s41467-023-44503-5)
Supplement: Supplementary file 5 — Reporting Summary [file 41467_2023_44503_MOESM5_ESM.pdf]

nature portfolio

Corresponding author(s): Jacob J Michaelson

Last updated by author(s): Nov 16, 2023

Reporting Summary

Nature Portfolio wishes to improve the reproducibility of the work that we publish. This form provides structure for consistency and transparency in reporting. For further information on Nature Portfolio policies, see our Editorial Policies and the Editorial Policy Checklist.

Statistics

For all statistical analyses, confirm that the following items are present in the figure legend, table legend, main text, or Methods section.

n/a	Confirmed	
☐	☒	The exact sample size (n) for each experimental group/condition, given as a discrete number and unit of measurement
☐	☒	A statement on whether measurements were taken from distinct samples or whether the same sample was measured repeatedly
☐	☒	The statistical test(s) used AND whether they are one- or two-sided<br>*Only common tests should be described solely by name; describe more complex techniques in the Methods section.*
☐	☒	A description of all covariates tested
☐	☒	A description of any assumptions or corrections, such as tests of normality and adjustment for multiple comparisons
☐	☒	A full description of the statistical parameters including central tendency (e.g. means) or other basic estimates (e.g. regression coefficient) AND variation (e.g. standard deviation) or associated estimates of uncertainty (e.g. confidence intervals)
☐	☒	For null hypothesis testing, the test statistic (e.g. F, t, r) with confidence intervals, effect sizes, degrees of freedom and P value noted<br>*Give P values as exact values whenever suitable.*
☒	☐	For Bayesian analysis, information on the choice of priors and Markov chain Monte Carlo settings
☒	☐	For hierarchical and complex designs, identification of the appropriate level for tests and full reporting of outcomes
☐	☒	Estimates of effect sizes (e.g. Cohen's d, Pearson's r), indicating how they were calculated

Our web collection on statistics for biologists contains articles on many of the points above.

Software and code

Policy information about availability of computer code

Data collection	No software was used to collect data in this study.
Data analysis	The model, NEUROeSTIMator, was trained with R (version 3.6.3) using the R package keras (version 2.3.0.0), with underlying dependencies of Python (version 3.7.8) and tensorflow (version 1.15.0). Software was installed from the conda-forge channel throuogh Miniconda3. NEUROeSTIMator is available at https://research-git.uiowa.edu/michaelson-lab-public/neuroestimator/ as a free R package with installation instructions and a tutorial.<br><br>R packages<br>biomaRt (version 2.46.3): gene identifier mapping.<br>scater (version 1.18.6): artificial downsampling of UMI counts for training data augmentation.<br>groupdata2 (version 1.4.1): sample allocation to cross-validation folds.<br>glmnet (version 4.1-6): fitting lasso-regularized linear models between NEUROeSTIMator output and electrophysiology features.<br>Seurat (version 4.0.1): normalization, integration, clustering, and visualization of Smart-seq/Patch-seq datasets.<br>fgsea (version 1.16.0): gene set enrichment analysis.<br>Rmagic (version 2.0.3): performance benchmarking comparison.<br><br>Other software:<br>IPFX (version 1.0.5): processing electrophysiology data.

For manuscripts utilizing custom algorithms or software that are central to the research but not yet described in published literature, software must be made available to editors and reviewers. We strongly encourage code deposition in a community repository (e.g. GitHub). See the Nature Portfolio guidelines for submitting code & software for further information.

Data

Policy information about availability of data

All manuscripts must include a data availability statement. This statement should provide the following information, where applicable:

- Accession codes, unique identifiers, or web links for publicly available datasets
- A description of any restrictions on data availability
- For clinical datasets or third party data, please ensure that the statement adheres to our policy

Spatial RNA-sequencing data, including gene expression measurements, tissue images, spot coordinates, and raw FASTQ files have been deposited in the Gene Expression Omnibus repository under the accession code "GSE201610 [https://www.ncbi.nlm.nih.gov/geo/query/acc.cgi?acc=GSE201610]".

GEO datasets used in this study can be accessed under the following accession codes:
“GSE111899 [https://www.ncbi.nlm.nih.gov/geo/query/acc.cgi?acc=GSE111899]”,
“GSE125068 [https://www.ncbi.nlm.nih.gov/geo/query/acc.cgi?acc=GSE125068]”,
“GSE55591 [https://www.ncbi.nlm.nih.gov/geo/query/acc.cgi?acc=GSE55591]”,
“GSE106678 [https://www.ncbi.nlm.nih.gov/geo/query/acc.cgi?acc=GSE106678]”,
“GSE137763 [https://www.ncbi.nlm.nih.gov/geo/query/acc.cgi?acc=GSE137763]”,
“GSE136656 [https://www.ncbi.nlm.nih.gov/geo/query/acc.cgi?acc=GSE136656]”,
“GSE102827 [https://www.ncbi.nlm.nih.gov/geo/query/acc.cgi?acc=GSE102827]”.

Other datasets used in this study can be accessed through the Allen Institute for Brain Science:
“Allen Cell Types Database [https://portal.brain-map.org/atlases-and-data/rnaseq]”,
“Mouse PatchSeq VIS [https://knowledge.brain-map.org/data/1HEYEW7GMUKWIQW37BO/summary]”,
“Human PatchSeq L2/3 [https://knowledge.brain-map.org/data/0R94W5U07IHCMVJ4TVK/summary]”.

Human research participants

Policy information about studies involving human research participants and Sex and Gender in Research.

Reporting on sex and gender	N/A
Population characteristics	N/A
Recruitment	N/A
Ethics oversight	N/A

Note that full information on the approval of the study protocol must also be provided in the manuscript.

Field-specific reporting

Please select the one below that is the best fit for your research. If you are not sure, read the appropriate sections before making your selection.

☒ Life sciences ☐ Behavioural & social sciences ☐ Ecological, evolutionary & environmental sciences

For a reference copy of the document with all sections, see nature.com/documents/nr-reporting-summary-flat.pdf

Life sciences study design

All studies must disclose on these points even when the disclosure is negative.

Sample size	Sample sizes were estimated based on previous studies in the field (Schurch, N.J. RNA 22, 839-851 (2016). No statistical tests were performed to predetermine sample size.
Data exclusions	Two samples were excluded from the analysis of spatial RNA-sequencing data (originally N=4 per group, final analysis used N=3 per group). These two samples were processed in succession, one from the control group and one from the trained SOR group. Both the predicted activity from our model and immediate early gene expression patterns of the two excluded samples matched the patterns observed in the opposite groups. We suspected these samples were mislabeled but we were unable to verify this.
Replication	Biological replicates were used for behavioral experiments. The RNAscope experiment was performed twice in cohorts of two animals per group. A minimum of two replicate sections from each brain were quantified.
Randomization	In each experimental batch, animals were chosen randomly based on their age.
Blinding	Brain sections were performed blindly to the training groups. Spatial gene expression (Visium) samples were sectioned and processed blind to

Blinding	the experimental groups. Similarly, RNAscope samples were also prepared, processed and imaged blind to the experimental groups.

Reporting for specific materials, systems and methods

We require information from authors about some types of materials, experimental systems and methods used in many studies. Here, indicate whether each material, system or method listed is relevant to your study. If you are not sure if a list item applies to your research, read the appropriate section before selecting a response.

Materials & experimental systems

n/a	Involved in the study
☒	☐ Antibodies
☒	☐ Eukaryotic cell lines
☒	☐ Palaeontology and archaeology
☐	☒ Animals and other organisms
☒	☐ Clinical data
☒	☐ Dual use research of concern

Methods

n/a	Involved in the study
☒	☐ ChIP-seq
☒	☐ Flow cytometry
☒	☐ MRI-based neuroimaging

Animals and other research organisms

Policy information about studies involving animals; ARRIVE guidelines recommended for reporting animal research, and Sex and Gender in Research

Laboratory animals	In this study, we used 2-4 months old C57BL/6J male mice purchased from The Jackson Laboratory.
Wild animals	The study did not include any wild animals
Reporting on sex	Male mice were used in this study
Field-collected samples	The study did not include any samples collected at the field
Ethics oversight	The experimental procedures followed the guidelines for animal care and use set forth by the National Institutes of Health, and they were approved by the Institutional Animal Care and Use Committee (IACUC) at the University of Iowa.

Note that full information on the approval of the study protocol must also be provided in the manuscript.
